# Preliminary observational study of metabonomics in patients with early and late-onset type 2 diabetes mellitus based on UPLC-Q-TOF/MS

**DOI:** 10.1038/s41598-023-41883-y

**Published:** 2023-09-04

**Authors:** Zhaohu Hao, Junxin Yao, Xiaoying Zhao, Ran Liu, Baocheng Chang, Hailin Shao

**Affiliations:** 1 Metabolic Disease Management Center of Endocrinology Department, Tianjin 4th Central Hospital, The 4th Center Clinical College of Tianjin Medical University, No.1 Zhongshan Road, Tianjin, 300140 China; 2 NHC Key Laboratory of Hormones and Development (Tianjin Medical University), Tianjin Key Laboratory of Metabolic Diseases, Tianjin Medical University Chu Hsien-I Memorial Hospital & Tianjin Institute of Endocrinology, Tianjin, 300134 China

**Keywords:** Diseases, Medical research

## Abstract

Non-targeted metabonomic techniques were used to explore changes in metabolic profiles of patients with early onset and late onset T2DM. Newly diagnosed early onset T2DM (EarT2DM) and late onset T2DM (LatT2DM) patients were recruited, and the matched age, sex, and low-risk population of diabetes mellitus were selected as the control group. 117 adults were recruited in the study, including 21 in EarT2DM group with 25 in corresponding control group (heaCG1), and 48 in LatT2DM group with 23 in corresponding control group (heaCG2). There were 15 relatively distinctive metabolic variants in EarT2DM group and 10 distinctive metabolic variants in LatT2DM group. The same changing pathways mainly involved protein, aminoacyl-tRNA biosynthesis, fatty acid biosynthesis, taurine metabolism, arginine biosynthesis, lysosome and mTOR signaling pathway. The independent disturbed pathways in EarT2DM included branched chain amino acids, alanine, aspartate and glutamate metabolism. The independent disturbed pathways in LatT2DM involved linoleic acid metabolism, biosynthesis of unsaturated fatty acids, arginine, proline metabolism and FoxO signaling pathway. T2DM patients at different diagnosed ages may have different metabolite profiles. These metabolic differences need to be further verified.

With the development of economy and the improvement of people's living standards, the incidence rate of diabetes mellitus (DM) is increasing year by year. The number of diabetic patients in China has increased rapidly, and the incidence of diabetes in China is 12.8% using the ADA criteria^1^. Type 2 diabetes mellitus (T2DM) is related to insulin resistance (IR) and islet β cell dysfunction, but the specific mechanism of its etiology and development is not completely clear. T2DM has always been considered to be a common disease in middle-aged and elderly people. The onset age of T2DM tends to be younger^2^. T2DM diagnosed age < 40 years is defined as early onset T2DM (EarT2DM), and T2DM with a diagnosed age ≥ 40 years is defined as late-onset T2DM (LatT2DM)^2^. The β cell function of EarT2DM is worse than that of LatT2DM^3^. An assessment of the β cell function using oral glucose tolerance tests and insulin release tests suggested that the annual decline in the β-cell function in patients with EarT2DM ranged between 20 and 35%^4^, while the annual decrease range for LatT2DM was only between 7 and 11%^5^. EarT2DM can result in more serious cardiovascular complications and risk of death^6^ with the life expectancy decreasing by 14 years in males and 16 years in females^7^. With the age of T2DM diagnosis delayed by 1 year, the risk of macrovascular complication, all-cause death and microvascular complication decreased by 3%, 4% and 5%, respectively^8^. EarT2DM patients are more likely to develop diabetic microvascular complications^9^. The metabolic disease management centre (MMC) of our hospital also conducted many epidemiological investigations on patients with diabetes. After balancing sex and disease factors, a positive correlation between early EarT2DM and the occurrence of diabetic retinopathy was found^10^.

EarT2DM patients may have stronger genetic susceptibilities and metabolic disorders^11,12^. EarT2DM is associated with genetic variants of β-cell function in the Chinese Han population^13^. Among the high-throughput analytical techniques, high performance liquid chromatography mass spectrometry (HPLC–MS) is used widely in the study of metabolic diseases^14^. In a study of patients with the metabolic syndrome using this technology, the central laboratory of our hospital identified a variety of representative changes in the metabolites^15^. High-throughput metabolomic techniques and technologies can provide vital insight into the preconditions (or risk factors) and pathophysiological pathways of T2DM^16^. A review of selected studies revealed a growing body of research demonstrating relationships between T2DM and metabolomic profifiles^17^, and researchers have examined how the existence and level of metabolites, such as amino acids, can facilitate understanding, diagnosing, and predicting the occurrence of T2DM^16^. Researchers concluded that an increased concentration of branched-chain amino acids is a reliable predictor of future insulin resistance among T2DM patients^18^.

Although a large number of studies have been done on metabolomics of DM, there are few studies on the metabolite characteristics of young and elderly diabetic patients. A follow-up study of postnatal diabetes mellitus in gestational diabetes has been carried out for 8 years. It was found that the initial metabolism of branched chain amino acids and arginine was significantly related to the development of diabetes in the later stage, but the average age of the subjects was 33 years old^19^. A study investigated the correlation between metabolic spectrum changes in adolescence and glucose metabolism in adulthood, and found that perturbed lipid metabolism as one of the earliest features of type 2 diabetes liability, alongside higher branched-chain amino acid and inflammatory levels in childhood as early as age 8 years, decades before the clinical onset of disease. But at the end of the observation, the subjects were about 25 years old^20^. Few studies focus on different characteristics of metabonomic of different onset ages of T2DM patients. The earlier the onset of diabetes, the higher the risk of complications. In this study, we investigated the characteristics of metabolite profiles in newly diagnosed type 2 diabetic patients with different diagnostic age (< 40 years and ≥ 50 years old) using non-targeted metabolomics technology, so as to prepare for further study the pathophysiological mechanism and complications of EarT2DM.

## Results

### General situation of the enrolled population

A total of 117 adults were recruited in the study, including 21 in the EarT2DM group with 25 in the corresponding control group (heaCG1), and 48 in the LatT2DM group with 23 in the corresponding control group (heaCG2). There were 14 men (66.7%) in the EarT2DM group, 13 in heaCG1 (52.0%), 21 in the LatT2DM group (43.8%), and 11 in the heaCG2 group (47.8%) (*P* > 0.05). The baseline data of the patients' age, blood triglyceride, cholesterol, low-density cholesterol, high-density cholesterol, body weight, and BMI were shown in Table 1.

**Table 1:** Comparison of baseline data between the recruited groups.

Items	EarT2DM (n = 21)	HeaCG1 (n = 25)	LatT2DM (n = 48)	HeaCG2 (n = 23)	P-value EarT2DM vs HeaCG1	P-value LatT2DM vs HeaCG2
Age (years)	34.0 ± 4.2	35.2 ± 5.3	58.9 ± 5.9	58.4 ± 9.0	0.384	0.796
Tg (mmol/l)	2.42 ± 0.65	1.87 ± 0.91	2.36 ± 1.02	1.83 ± 0.78	0.026	0.034
Tc (mmol/l)	5.02 ± 1.06	4.94 ± 0.68	5.38 ± 1.29	5.11 ± 0.89	0.765	0.383
HDL-c (mmol/l)	1.21 ± 0.45	1.20 ± 0.29	1.10 ± 0.28	1.32 ± 0.36	0.960	0.012
LDL-c (mmol/l)	3.31 ± 0.88	3.25 ± 0.81	3.51 ± 1.11	3.43 ± 0.85	0.813	0.772
Weight (kg)	81.9 ± 12.8	73.6 ± 13.4	81.0 ± 10.3	74.6 ± 16.6	0.037	0.045
BMI (kg/m^2^)	28.9 ± 3.5	25.7 ± 5.6	27.5 ± 4.1	25.2 ± 4.2	0.026	0.025

*EarT2DM* Early-onset T2DM, *HeaCG1* Healthy Control Group 1, *LatT2DM* Late-onset T2DM, *HeaCG2* Healthy Control Group 2, *Tg* triglyceride, *Tc* total cholesterol, *HDL-c* high-density cholesterol, *LDL-c* low-density cholesterol, *BMI* body mass index.

**Figure 1:**
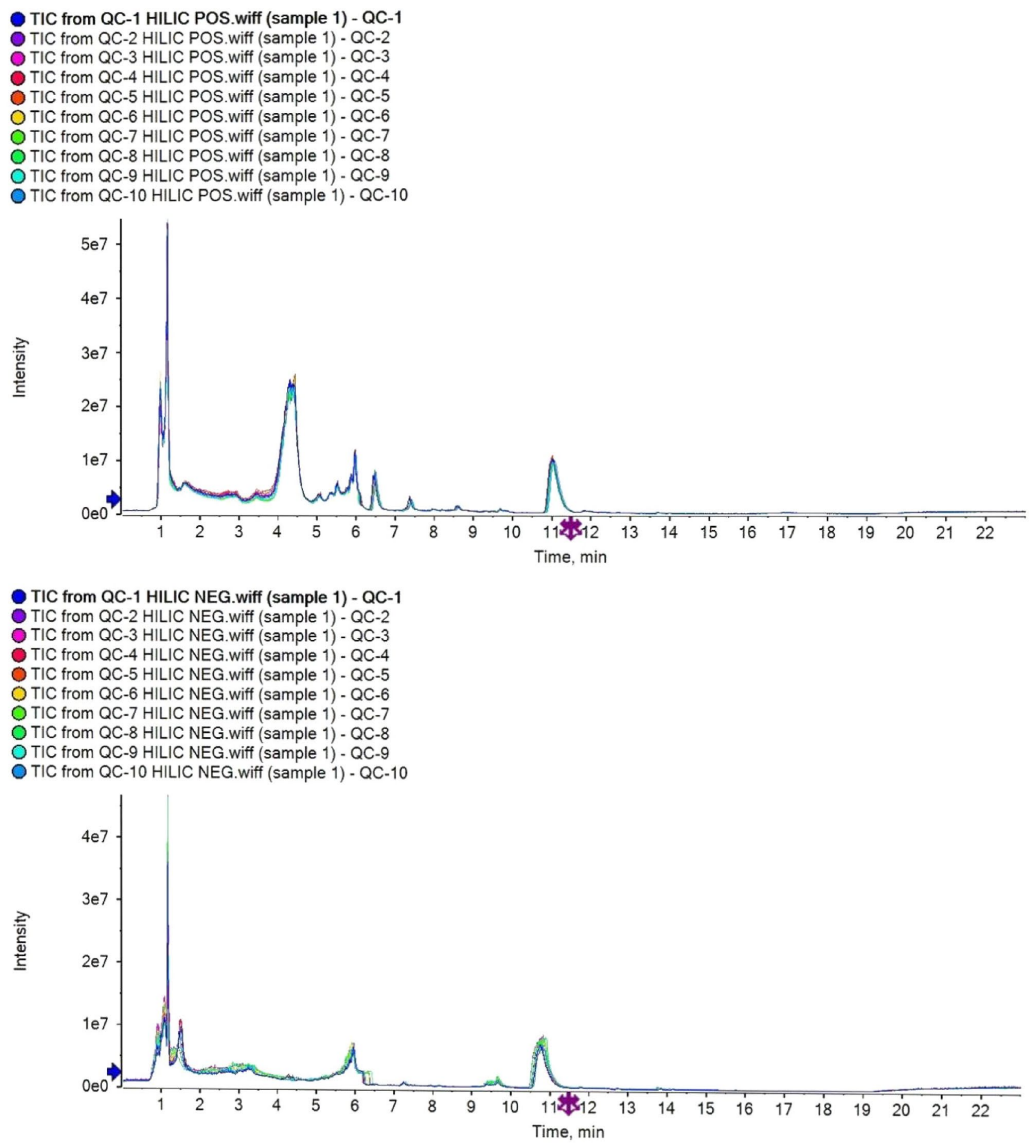
Total ion chromatogram.

For the untargeted metabolomic analyses, 117 plasma samples were examined. In the metabolic profiling, 2553 positive-mode features and 3358 negative-mode features were identified and used in the Pareto scaling. The total ion chromatogram was shown in Fig. 1.

### Comparison of metabonomics results between EarT2DM group and heaCG1

**Figure 2:**
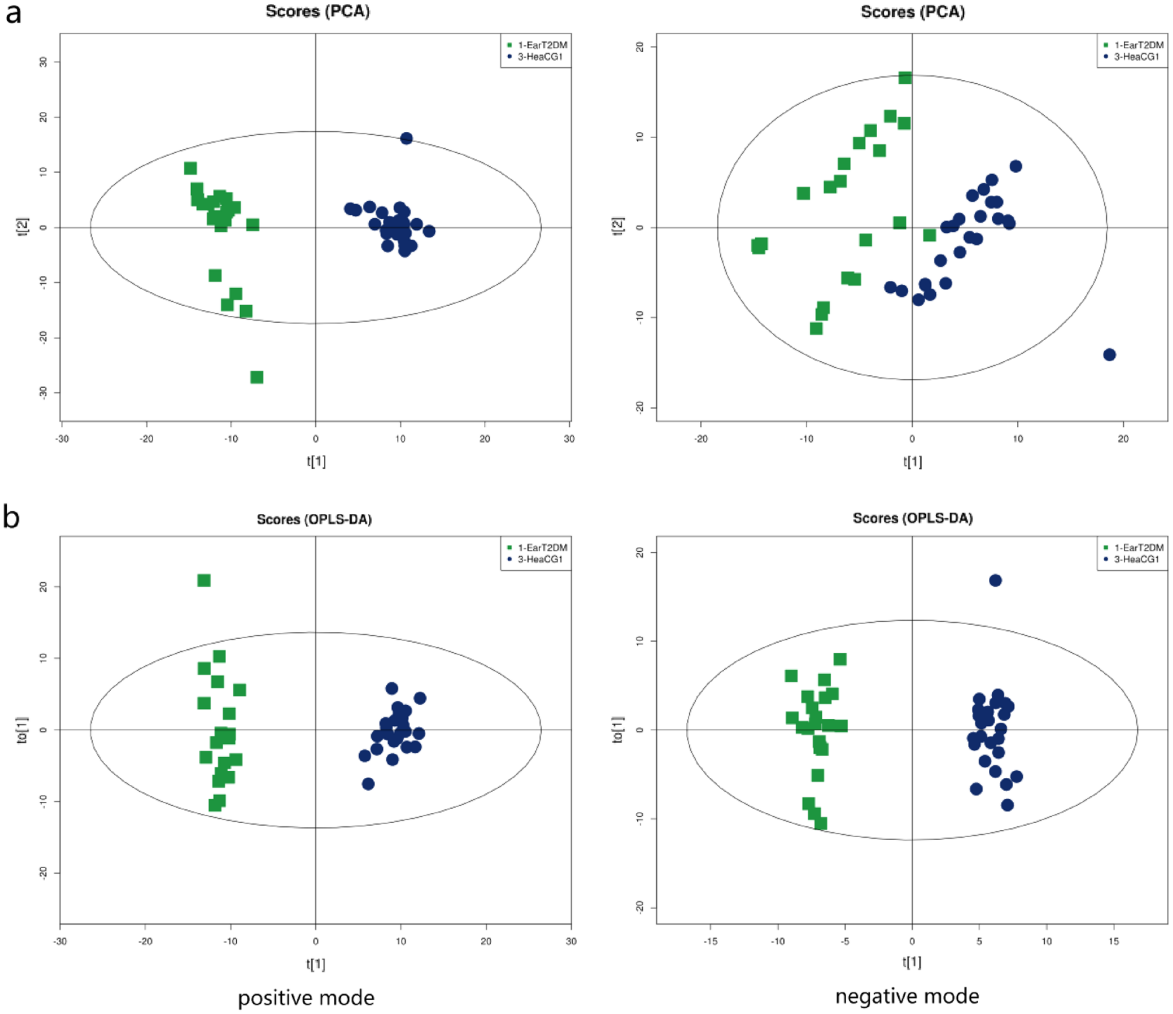
Comparison of metabonomics results between EarT2DM group and heaCG1 (PCA and OPLS-DA). (**a**) Principal Component Analysis (PCA) score plots; (**b**) Orthogonal partial least-squares discriminant analysis(OPLS-DA) score plots.

The results of the PCA and OPLS-DA in the EarT2DM and control groups were shown in Fig. 2. The PCA results showed R2X > 0.5. The two OPLS-DA models were evaluated using the R2Y and Q2 parameters. The values of R2Y were 0.981 and Q2 0.969 in the positive mode, and R2Y 0.980 and Q2 0.924 in the negative mode. The samples of the EarT2DM group were separated intelligibly from the control samples in both the positive and negative modes, and the obvious separation suggested that there was pronounced metabolic differences at an overview level.

**Figure 3:**
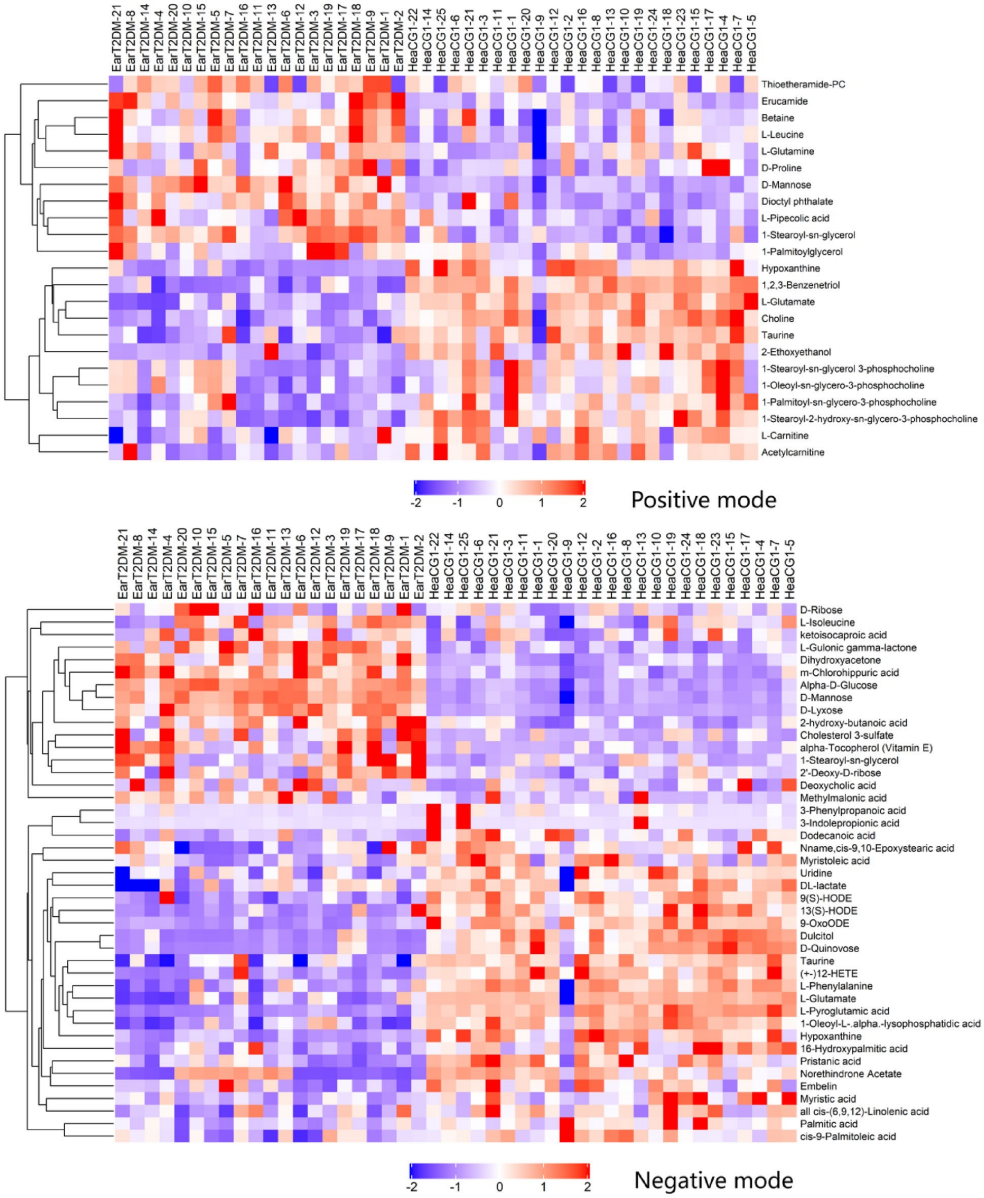
Comparison of metabonomics cluster plot between EarT2DM group and heaCG1.

The differential metabolites between the groups were filtered using the multivariate and univariate statistical significance criteria (VIP > 1 and p < 0.05). Figure 3 showed the hierarchical clustering results of metabolites with significant differences in the EarT2DM group. In total, 66 identified metabolites showed significant differences between the two groups, including 23 species in positive mode and 43 species in negative mode.

### Comparison of metabonomics results between LatT2DM group and heaCG2

**Figure 4:**
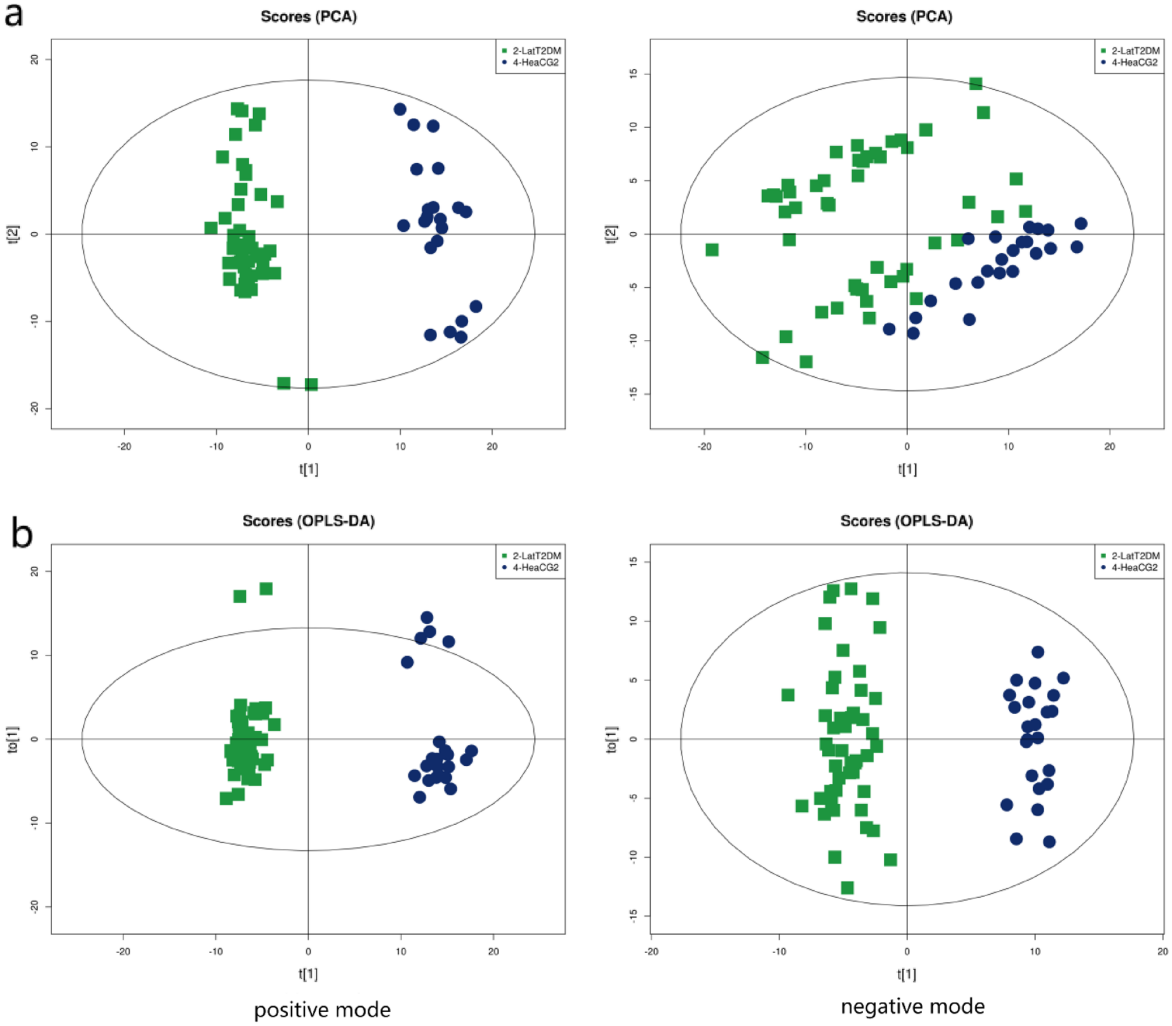
Comparison of metabonomics results between LatT2DM group and heaCG2 (PCA and OPLS-DA). (**a**) Principal Component Analysis (PCA) score plots; (**b**) Orthogonal partial least-squares discriminant analysis(OPLS-DA) score plots.

The results of the PCA and OPLS-DA in the LatT2DM and control groups were shown in Fig. 4. The PCA results showed R2X > 0.5. The two OPLS-DA models were evaluated using the R2Y and Q2 parameters. The values of R2Y were 0.979 and Q2 0.958 in the positive mode and R2Y 0.981 and Q2 0.937 in the negative mode. The samples of the LatT2DM group were separated intelligibly from the control samples in both positive and negative modes, and the obvious separation suggested that there were pronounced metabolic differences at an overview level.

**Figure 5:**
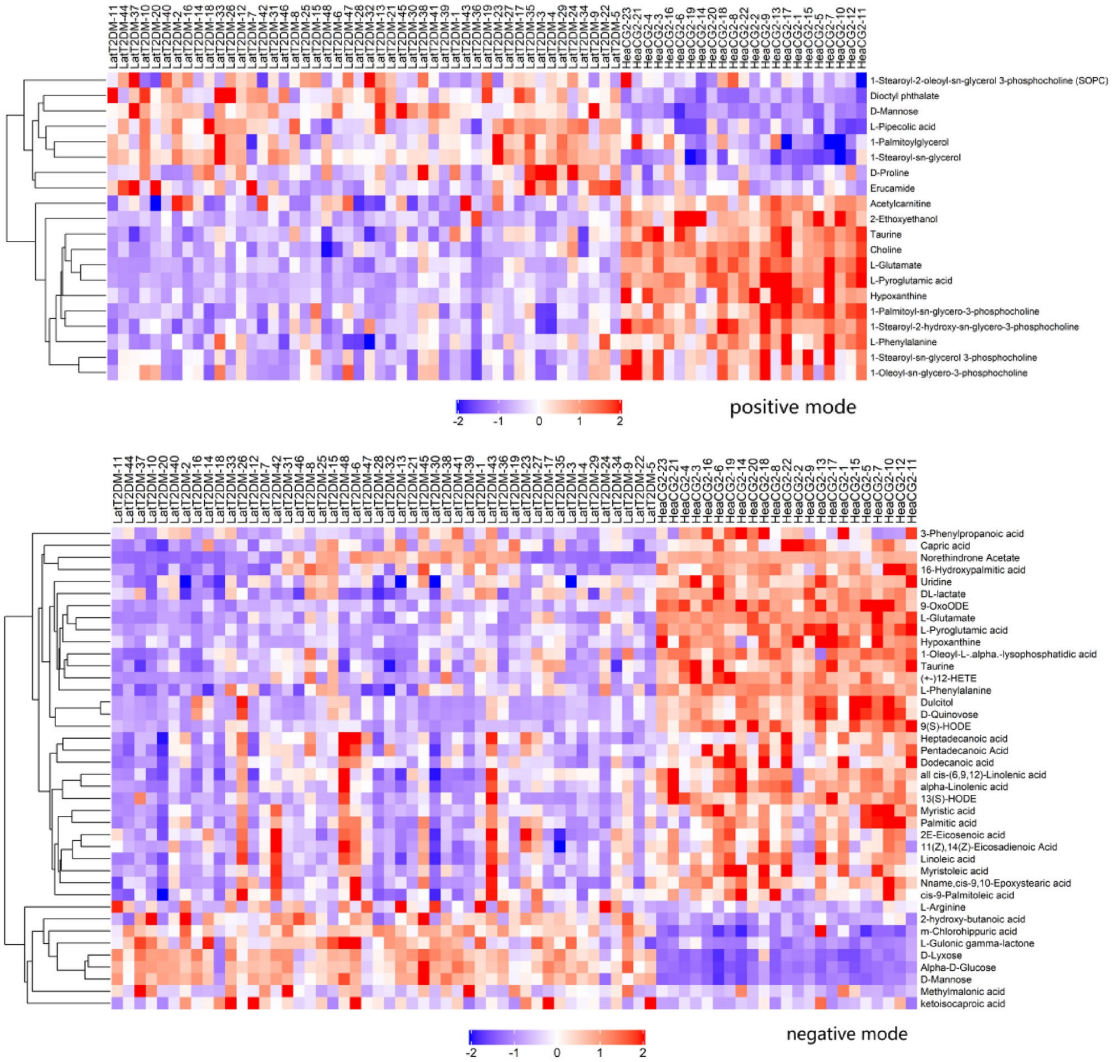
Comparison of metabonomics cluster plot between LatT2DM group and heaCG2.

Figure 5 showed the hierarchical clustering results of the metabolites with significant differences in the LatT2DM group. In total, 60 identified metabolites showed significant differences, including 20 species in the positive mode and 40 species in negative mode.

### Further analysis of differential metabolites between EarT2DM group and LatT2DM group

The differentiated between the groups were filtered using multivariate and univariate statistical significance criteria (VIP > 1 and p < 0.05). Compared with the corresponding healthy control groups, 12 metabolites increased and 30 decreased in the T2DM groups. There were 15 relatively distinctive metabolic variants in the EarT2DM group. The significantly elevated metabolites included l-leucine, betaine, thioetheramide-PC, l-glutamine, dihydroxyacetone, vitamin E, cholesterol 3-sulfate, 2'-deoxy-d-ribose, l-isoleucine, methylmalonic acid and deoxycholic acid. Metabolites with significantly decreased expression included 1,2,3-benzenetriol, l-carnitine, pristanic acid and embelin. There were 10 relatively distinctive metabolic variants in the LatT2DM group. The elevated metabolites included d-proline, and l-arginine. The decreased metabolites included 3-phenylpropanoic acid, alpha-linolenic acid, linoleic acid, capric acid, pentadecanoic acid, 2E-Eicosenoic acid, 11(Z), 14(Z)-Eicosadienoic acid, and heptadecanoic acid. The detailed information on these metabolites was shown in Table 2.

**Table 2:** Relative difference of metabolites between EarT2DM and LatT2DM groups.

Metabolites	ESI^+/−^	EarT2DM vs HeaCG1			LatT2DM vs HeaCG2		
		VIP	FC	P-value	VIP	FC	P-value
d-Mannose	+	3.07	2.08	< 0.001	2.98	2.00	< 0.001
l-Glutamate	−	1.78	0.50	< 0.001	1.94	0.40	< 0.001
Choline	+	3.92	0.62	< 0.001	4.28	0.60	< 0.001
1-Stearoyl-sn-glycerol	+	1.76	1.63	< 0.001	2.15	1.68	< 0.001
1-Stearoyl-2-hydroxy-sn-glycero-3-phosphocholine	+	6.88	0.62	< 0.001	6.97	0.59	< 0.001
Hypoxanthine	+	2.74	0.37	< 0.001	3.24	0.32	< 0.001
2-Ethoxyethanol	+	1.10	0.46	< 0.001	1.59	0.38	< 0.001
Taurine	+	1.02	0.69	< 0.001	1.44	0.57	< 0.001
Dioctyl phthalate	+	1.05	1.64	< 0.001	1.33	1.94	< 0.001
Erucamide	+	2.01	2.43	< 0.001	1.48	1.63	0.019
l-Pipecolic acid	+	1.04	1.81	< 0.001	1.04	1.82	< 0.001
1-Palmitoyl-sn-glycero-3-phosphocholine	+	8.53	0.71	< 0.001	10.35	0.64	< 0.001
Acetylcarnitine	+	2.14	0.73	0.002	1.99	0.72	< 0.001
1-Oleoyl-sn-glycero-3-phosphocholine	+	2.78	0.78	0.005	2.50	0.75	< 0.001
1-Stearoyl-sn-glycerol 3-phosphocholine	+	1.46	0.78	0.006	1.82	0.69	< 0.001
1-Palmitoylglycerol	+	1.05	1.35	0.020	1.57	1.15	0.004
Alpha-D-Glucose	−	10.92	2.32	< 0.001	9.83	2.22	< 0.001
l-Pyroglutamic acid	−	3.54	0.41	< 0.001	3.12	0.39	< 0.001
d-Lyxose	−	1.28	2.58	< 0.001	1.10	2.15	< 0.001
d-Quinovose	−	4.53	0.17	< 0.001	3.46	0.25	< 0.001
Dulcitol	−	2.99	0.22	< 0.001	2.26	0.29	< 0.001
m-Chlorohippuric acid	−	2.19	1.50	< 0.001	1.93	1.41	< 0.001
9-OxoODE	−	1.99	0.32	< 0.001	1.73	0.31	< 0.001
(+ −)12-HETE	−	4.34	0.28	< 0.001	3.76	0.26	< 0.001
l-Gulonic gamma-lactone	−	3.98	2.42	< 0.001	3.81	2.80	< 0.001
1-Oleoyl-L-.alpha.-lysophosphatidic acid	−	1.65	0.48	< 0.001	1.55	0.42	< 0.001
9(S)-HODE	−	1.13	0.44	< 0.001	1.22	0.30	< 0.001
13(S)-HODE	−	2.30	0.47	< 0.001	2.16	0.40	< 0.001
L-Phenylalanine	−	2.90	0.64	< 0.001	2.54	0.62	< 0.001
2-hydroxy-butanoic acid	−	2.51	1.79	< 0.001	2.11	1.83	< 0.001
16-Hydroxypalmitic acid	−	2.40	0.55	< 0.001	2.43	0.51	< 0.001
Uridine	−	1.09	0.81	< 0.001	1.07	0.73	< 0.001
Norethindrone Acetate	−	7.15	0.51	< 0.001	6.93	0.48	< 0.001
Myristic acid	−	4.48	0.62	0.001	4.85	0.54	< 0.001
Cis-9-Palmitoleic acid	−	4.10	0.76	0.001	2.98	0.77	0.008
Dodecanoic acid	−	2.26	0.61	0.002	1.75	0.59	< 0.001
DL-lactate	−	4.27	0.82	0.003	4.92	0.70	< 0.001
Palmitic acid	−	10.78	0.80	0.003	12.91	0.75	< 0.001
Myristoleic acid	−	1.90	0.63	0.004	2.37	0.52	< 0.001
All cis-(6,9,12)-Linolenic acid	−	2.36	0.84	0.010	3.94	0.64	< 0.001
Nname,cis-9,10-Epoxystearic acid	−	1.42	0.77	0.029	1.76	0.58	< 0.001
Ketoisocaproic acid	−	8.59	1.32	0.048	1.24	1.41	0.019
1,2,3-Benzenetriol	+	1.02	0.39	< 0.001	–	–	–
l-Leucine	+	2.02	1.27	< 0.001	–	–	–
Betaine	+	1.02	1.12	0.004	–	–	–
l-Carnitine	+	1.49	0.82	0.006	–	–	–
Thioetheramide-PC	+	4.49	1.49	0.023	–	–	–
l-Glutamine	+	1.03	1.09	0.040	–	–	–
Dihydroxyacetone	−	1.03	2.11	< 0.001	–	–	–
Pristanic acid	−	1.19	0.65	< 0.001	–	–	–
Alpha-Tocopherol (Vitamin E)	−	1.73	1.75	< 0.001	–	–	–
Cholesterol 3-sulfate	−	5.25	1.74	< 0.001	–	–	–
2'-Deoxy-d-ribose	−	4.28	1.55	0.002	–	–	–
Embelin	−	1.23	0.70	0.013	–	–	–
l-Isoleucine	−	2.02	1.24	0.016	–	–	–
Methylmalonic acid	−	1.34	1.58	0.025	1.16	1.55	0.054
Deoxycholic acid	−	1.79	1.61	0.032	–	–	–
d-Ribose	−	1.30	1.40	0.058	–	–	–
3-Indolepropionic acid	−	4.96	0.06	0.096	–	–	–
SOPC	+	1.12	1.14	0.074	–	–	–
d-Proline	+	1.33	1.14	0.087	1.60	1.21	0.008
3-Phenylpropanoic acid	−	1.65	0.20	0.099	1.17	0.45	< 0.001
Alpha-Linolenic acid	−	–	–	–	7.78	0.53	< 0.001
Linoleic acid	−	–	–	–	19.72	0.67	< 0.001
Capric acid	−	–	–	–	1.24	0.67	< 0.001
Pentadecanoic acid	−	–	–	–	1.23	0.71	< 0.001
l-Arginine	−	–	–	–	1.01	2.12	0.006
2E-Eicosenoic acid	−	–	–	–	1.29	0.79	0.017
11(Z),14(Z)-Eicosadienoic acid	−	–	–	–	1.40	0.83	0.025
Heptadecanoic acid	−	–	–	–	1.21	0.82	0.026

*EarT2DM* Early-onset T2DM, *LatT2DM* Late-onset T2DM, *VIP* variable importance in the projection, *FC* Fold change, *ESI* Electrospray ionization, *SOPC* 1-Stearoyl-2-oleoyl-sn-glycerol 3-phosphocholine.

**Figure 6:**
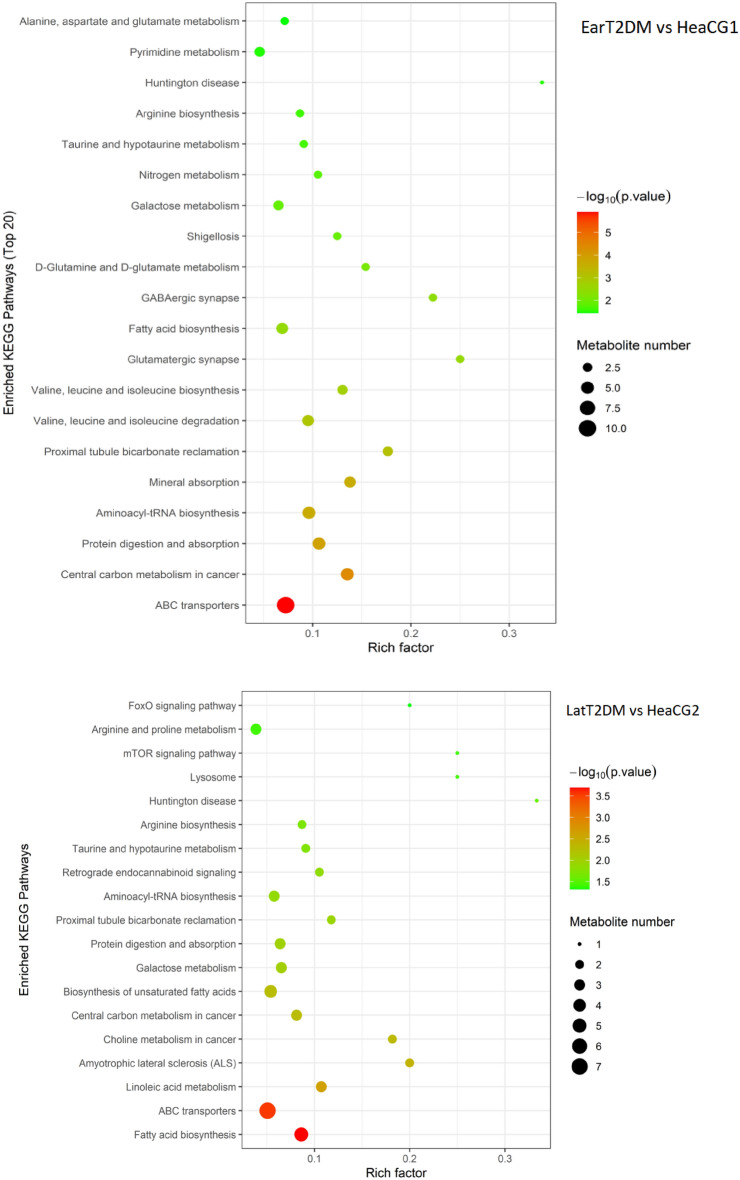
Enriched KEGG Pathways (Enrichment Bubble).

KEGG was used to gain further understanding of the metabolic disturbances in the EarT2DM and LatT2DM groups. The significantly perturbed pathways were shown in Fig. 6^21–23^. Compared with HeaCG1, EarT2DM had 22 significantly altered pathways, mainly including protein, fatty acid, amino acid biosynthesis and degradation. LatT2DM had 20 significantly altered pathways, mainly including fatty acid, linoleic acid and galactose metabolism. The same changing pathways mainly involved protein, aminoacyl-tRNA biosynthesis, fatty acid biosynthesis, taurine metabolism, arginine biosynthesis, lysosome and mTOR signaling pathway.

Excluding the same metabolic pathways, the independent disturbed pathways in EarT2DM included branched chain amino acids, alanine, aspartate and glutamate metabolism. The independent disturbed pathways in LatT2DM involved linoleic acid metabolism, biosynthesis of unsaturated fatty acids, arginine, proline metabolism and FoxO signaling pathway.

## Discussion

### EarT2DM and LatT2DM patients had metabolic spectrum changes

In this study, it was found that compared with the corresponding control group, they had common metabolic spectrum changes, but also had their own relatively specific changes. The same changing pathways mainly involved protein, aminoacyl-tRNA biosynthesis, fatty acid biosynthesis, taurine metabolism, lysosome and mTOR signaling pathway. In this study, they also had relatively specific metabolic changes. Combined with KEGG analysis, the difference of amino acid metabolism was obvious in the metabolic spectrum of the two groups. These pathways mainly involved branched chain amino acids, GABA, alanine, aspartate and glutamate metabolism in EarT2DM group. The mainly disturbed pathways in LatT2DM group included linoleic acid metabolism, biosynthesis of unsaturated fatty acids, arginine and proline metabolism.

### Leucine and isoleucine levels in EarT2DM group were higher

Branched chain amino acids, including valine, leucine and isoleucine, are essential amino acids.The study showed that l-leucine and l-isoleucine levels increased in patients with early onset T2DM. A cohort of 2422 patients without diabetes who underwent physical examinations between 1991 and 1995 was studied. Fasting serum samples were taken at baseline for metabonomics. Metabolic changes were found to occur in the early stages of diabetes mellitus. During the 12-year follow-up period, 201 patients developed type 2 diabetes. Isoleucine, leucine, valine, tyrosine and phenylalanine were significantly higher, and the analysis of their metabolites could have important predictive value for new-onset diabetes mellitus^24^. Varieties of amino acids, especially leucine, are important regulators of the mTORC1 signal^25^. The increased level of branched chain amino acids in plasma over a long period of time may lead to the over-activation of mTOR signalling and may lead to early β cell dysfunction and destruction^26^. Branched chain amino acids can also promote glucose uptake in the liver and skeletal muscle and promote glycogen synthesis through the phosphatidylinositol 3-kinase or protein kinase C pathway^27^. Branched-chain amino acids may also affect glucose and lipid metabolism through the intestinal flora^28^. In a study of the American population, the predictive value of phenylalanine and valine was better than the other three amino acids^24^. Studies have shown that branched chain amino acids are the strongest predictors of the progression and prognosis of metabolic diseases such as diabetes and obesity, which are stronger than those related to lipid metabolism^29^. In the study we found that leucine and isoleucine levels in early onset T2DM patients were significantly higher compared to those in the control group, and this phenomenon was not observed in late-onset T2DM.

### l-glutamine (Gln) levels were increased in EarT2DM group

l-glutamine is one of the most abundant amino acids in the human body. It is a conditional essential amino acid that plays a vital role in nitrogen exchange, intermediate metabolism, immunity, and pH homeostasis^30^. Studies have found that plasma Gln is negatively correlated with body mass index, blood pressure, blood triglyceride and insulin levels, and positively correlated with high-density lipoprotein^31^. Gln circulation pathway may stimulate the pancreas by promoting the release of glucagon like peptide 1 and the activation of glucose transporter β cellular insulin release and insulin gene transcription^32^. Metabonomic analysis of human adipocytes showed that Gln attenuated glycolysis and reduced the level of uridine diphosphate N-acetylglucosamine (UDP GlcNAc). UDP GlcNAc is an o-junction mediated by O-GlcNAc transferase β-N-acetylglucosamine post-translational modified substrate is closely related to the existence of proinflammatory transcriptional response of human adipocytes^33^. Glutamine reduces macrophage infiltration in white adipose tissue and reduces inflammatory responses^34^. Glutamine metabolism in macrophage polarisation regulation function or by manipulating macrophage polarisation to prevent or improve obesity or type 2 diabetes^35^. Animal studies have shown that L-Gln supplementation can improve insulin resistance in liver and muscle of obese mice^36^.

### l-arginine was increased significantly in LatT2DM group

l-arginine is a functional amino acid and a precursor of nitric oxide. It plays an important role in animal maintenance, reproduction, growth, anti-aging and immunity. More and more clinical evidence shows that dietary supplementation of l-arginine can reduce obesity, lower arterial blood pressure, antioxidant and endothelial dysfunction, thereby relieving T2DM^37^. The signal pathway of l-arginine beneficial effect may include l-arginine-nitric oxide pathway, through which cellular signal proteins can be activated. More and more studies have shown that l-arginine may have the potential to prevent and/or alleviate T2DM by restoring insulin sensitivity in vivo^37^. The observed safe level for oral administration of Arg is ∼20 g/d^38^. Arginine and glycine increased the risk of T2DM in the western countries’ subgroup^39^. In addition, the slight improvements in T2DM prediction beyond traditional risk factors were observed when these metabolites were added in the predictive analysis^39^.

### Alpha-linolenic acid and linoleic acid were decreased in LatT2DM group

Linoleic acid can be metabolized to n-6 via desaturase, resulting via the biosynthesis of gamma-linolenic acid, dihomo-gamma-linolenic acid, and finally arachidonic acid^40^. The largest amount of arachidonic acid is found in phospholipid membranes, competing with n-3 acids for metabolism and with their products for receptors^40^. Linoleic acid as an unsaturated fatty acid linoleic acid has been proved to be beneficial to human body, including lipid-lowering, anti atherosclerosis, anti diabetes and anti-inflammatory effects^41,42^.

### The decrease of pristanic acid level was in EarT2DM group

Pristanic acid originates mainly from the endogenous transformation of phytanoic acid in the liver and acts as a peroxisome proliferator activated receptor (PPAR)- α, PPAR-γ, and the natural ligand of retinoid X receptor, which is involved in peroxisome and mitochondrial function, oxidative stress, inflammatory pathway, cell signal transduction, glucose/energy metabolism, and microbial effects^43^, and can promote the expression of glucose carriers on the cell membrane and improve insulin sensitivity^44^. In this study, the decrease of pristanic acid level in patients with early onset T2DM needs further research to confirm whether it is related to insulin resistance in patients with early onset T2DM.

There were also other characteristics in the metabolic spectrum between patients with early onset T2DM and late-onset T2DM compared to their healthy control groups. The findings of this study had several limitations. Firstly, this was a single-centre investigation. Secondly, the research object in this study was mainly the urban population of Tianjin, China. Thirdly, the study was only a preliminary study with a small sample size, and the metabolites and pathways involved need to be further verified.

In summary, patients with type 2 diabetes at different diagnosed ages may have different metabolite profiles. In the metabolism of amino acids and their derivatives, patients with early onset T2DM may have more level of l-glutamine and branched chain amino acids (especially leucine and isoleucine). For newly diagnosed T2DM patients over 50 years of age, there may be metabolic changes in arginine level and the decrease of unsaturated fatty acids. Therefore, it is necessary to consider the diagnosed age factor when using metabolic indicators as predictor of T2DM. Further studies are needed to determine whether the higher risk of complications in early-onset type 2 diabetes is related to its specific metabolic profile.

## Methods and materials

### Patients

**Figure 7:**
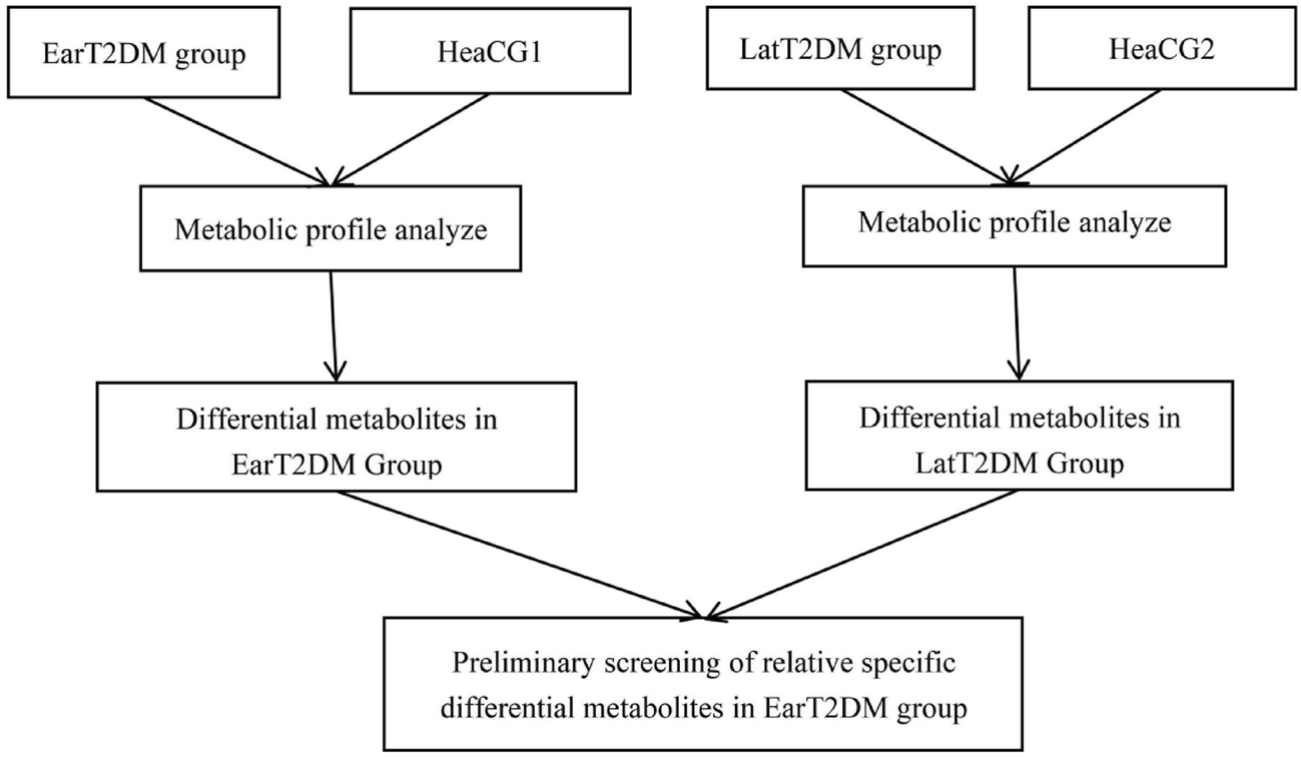
Flow chart of the study. *EarT2DM* Early-onset T2DM, *HeaCG1* Healthy Control Group 1, *LatT2DM* Late-onset T2DM, *HeaCG2* Healthy Control Group 2.

Newly diagnosed T2DM patients treated at the MMC of the Tianjin 4th Central Hospital from April to August 2018 were recruited as the research subjects, and healthy people were recruited as the control group at the same time. In this study, healthy people were referred to as volunteers who had no diabetes and had a low risk of diabetes when they entered the group (China Diabetes Risk Score < 25)^45^. We collected information on the patient’s name, sex, smoking and drinking history, family history of diabetes, waist circumference, age, diabetes course, and blood pressure from the MMC system. The body mass index (BMI) was calculated and recorded according to the height and body weight. Blood was taken in a fasting state for the detection of plasma glucose and blood lipids. The patients were examined for proteinuria, the fundus was photographed, and serum samples were taken for metabolomic analysis. Flow chart of the study was shown in Fig. 7.

### Ethical statement

The clinical study protocol was approved by the Institutional Review Board (IRB) of Tianjin 4th Central Hospital, and all steps were conducted in accordance with the principles of the World Medical Association Declaration of Helsinki (trial registration code: ChiCTR2000037652). The IRB approved the collection and use of patient records according to the regulations for clinical trials in humans (IRB approval no.2017-SZXLL017). Written informed consent was obtained from each patient, and serum samples were collected during medical examinations.

### Inclusion and exclusion criteria

Due to the occult onset of T2DM, there might be a large gap between the time of diagnosis and the time of real onset^46^. In this study, the diagnosed age of LatT2DM was ≥ 50 years, and the diagnosed age of EarT2DM was < 40 years. In order to avoid the influence of drugs on the metabolic spectrum results, the subjects did not take any drugs or health products in the recent period (within 1 month). Each participant recently had regular rest and diet, and there was no stress such as excessive exercise, trauma and emotional excitement. All diabetic patients were screened for insulin antibodies, glutamic acid decarboxylase antibodies and islet cell antibodies, all of which were negative. The inclusion criteria: (a) The diagnosis of T2DM was based on the 2017 Chinese diabetes treatment guidelines, as fasting peripheral blood glucose (FPG) ≥ 7.0 mmol/l, postprandial blood glucose ≥ 11.1 mmol/l at 2 h, or a confirmed diagnosis as type 2 diabetes by hospitals with Grade 2 or above^1^. (b) The duration of diabetes was less than 1 year. (c) Patients did not have diabetic microvascular complications, such as proteinuria or diabetic retinopathy. (d) Each patient volunteered to participate in the study.

The exclusion criteria were as follows: (a) type 1 and other special types of diabetes, gestational diabetes, or diabetes mellitus with pregnancy; (b) patients with severe mental illness and unclear consciousness; and (c) patients with active tuberculosis and other infectious diseases.

Group description: According to the age at diabetes diagnosis, the study divided patients with diabetes into EarT2DM and LatT2DM groups, and matched healthy individuals with age and sex as corresponding healthy control groups. They were successively named healthy control group 1 (HeaCG1) and healthy control group 2 (HeaCG 2).

### Ethics approval and consent to participate

The IRB approved the collection and use of patient records according to the regulations for clinical trials in humans (IRB approval NO.2017-SZXLL017). All steps were conducted in accordance with the principles of the World Medical Association Declaration of Helsinki (trial registration code: ChiCTR2000037652). Written informed consent was obtained from all the participants.

## UPLC-Q-TOF/MS analysis^15^

### Sample collection and preparation

Serum collection: Blood samples were processed by spinning the blood at 3000 rpm for 10 min at 4 °C and freezing at − 80 °C prior to analysis. The samples were thawed at 4 °C and 100 μL aliquots were mixed with 400 μL of cold methanol/acetonitrile (1:1, v/v) to remove the protein. The mixture was centrifuged for 20 min at 14,000 × *g* and 4 °C. The supernatant was then dried using a vacuum centrifuge. For the UPLC-MS analysis, the samples were redissolved in 100 μL of cold methanol/acetonitrile (1:1, v/v).

### Requirements, operation and analysis of experimental platform

Platform requirements: triple TOF 5600 + mass spectrometer (AB SCIEX), Agilent 1290 infinity LC ultra high pressure liquid chromatograph (Agilent), chromatographic column: waters, acquity UPLC beh amide 1.7 μm, 2.1 mm × 100 mm column.

Chromatographic conditions and process: the samples were separated by Agilent 1290 infinity LC ultra high performance liquid chromatography system (UHPLC) HILIC column; Column temperature 25 ℃; Flow rate: 0.3 ml / min; Injection volume 2 μL; Mobile phase composition a: water + 25 mM ammonium acetate + 25 mm ammonia, B: acetonitrile; The gradient elution procedure is as follows: 0–1 min, 95% B; 1–14 min, B changes linearly from 95 to 65%; 14–16 min, B changes linearly from 65 to 40%; 16–18 min, B maintained at 40%; 18–18.1 min, B changes linearly from 40 to 95%; 18.1–23 min, B maintained at 95%; During the whole analysis process, the samples were placed in a 4 ℃ automatic sampler. In order to avoid the influence caused by the fluctuation of instrument detection signal, the random sequence is used for continuous analysis of samples.

Mass spectrum condition setting and process: The ESI source conditions were set as follows: Ion Source Gas1 (Gas1) as 60, Ion Source Gas2 (Gas2) as 60, curtain gas (CUR) as 30, source temperature, 600 °C; and IonSpray voltage floating (ISVF) ± 5500 V. On MS acquisition, the instrument was set to acquire over the m/z range 60–1000 Da, and the accumulation time for TOF MS scan was set at 0.2 s/spectra. In the auto MS/MS acquisition, the instrument was set to acquire over the m/z range of 25–1000 Da, and the accumulation time for the product ion scan was set at 0.05 s/spectra. The product ion scan was acquired using information-dependent acquisition (IDA) with a high sensitivity mode. The parameters were set as follows: the collision energy (CE) was fixed at 35 V with ± 15 eV; declustering potential (DP),60 V ( +), and − 60 V ( −), excluding isotopes within 4 Da, candidate ions to monitor per cycle: 6.

In the HILIC separation, samples were analysed using a 2.1 mm × 100 mm ACQUIY UPLC BEH 1.7 µm column (Waters, Ireland). In both ESI positive and negative modes, the mobile phase contained 25 mM ammonium acetate and 25 mM ammonium hydroxide in water and B = acetonitrile. The gradient was 95% eluent B for 1 min and was linearly reduced to 65% in 14 min, and then reduced to 40% in 2 min, maintained for t2 min, and then increased to 95% in 0.1 min, with a 5 min re-equilibration period. The gradients were at a flow rate of 0.3 mL/min, and the column temperatures were kept constant at 25 °C. A 2 µL aliquot of each sample was injected. During the analysis, quality control samples (QC) were inserted into the sample queue to monitor and evaluate the stability of the system and the reliability of the experimental data.

### Metabolite data analysis

The raw MS data (wiff. scan files) were converted to mzXML files using the Proteo Wizard MS Convert before importing into freely available XCMS software. For peak picking, the following parameters were used: centre-wave m/z = 25 ppm, peak width = c (10, 60), and prefilter = c (10, 100). For peak grouping, bw = 5, mzwid = 0.025, and minfrac = 0.5. In the extracted ion features, only the variables with more than 50% of the nonzero measurement values in at least one group were retained. The identification of the metabolites using MS/MS with an in-house database was established using available authentic standards. After the normalisation to total peak intensity, the processed data were uploaded before importing into SIMCA-P (version 14.1, Umetrics, Umea, Sweden), where the data were subjected to multivariate data analyses, including Pareto-scaled principal component analyses (PCAs) and orthogonal partial least-squares discriminant analyses (OPLS-DAs). The variable importance in the projection (VIP) value of each variable in the PLS-DA model was calculated to indicate its contribution to the classification. Metabolites with a VIP value > 1 were further subjected to Student’s t-tests at the univariate level to measure the significance of each metabolite, and *P* values less than 0.05, were considered to be statistically significant. The hierarchical cluster algorithm was used to cluster the differentially expressed proteins among groups, and the data were displayed in the form of a heat map.

The hierarchical cluster algorithm was used to cluster the differentially expressed proteins among groups, and the data were displayed in the form of a heat map. Taking the KEGG pathway as a unit and taking the metabolic pathway participated by this species or species with close genetic relationships as the background, the significance level of metabolite enrichment of each pathway was analysed and calculated using the Fisher's exact test to make a preliminary determination of the significantly affected metabolic and signal transduction pathways.

### Statistical analysis

Statistical Program for Social Sciences software (version 20.0; SPSS, Inc., Chicago, IL, USA) was used for data collation and analysis. The Kolmogorov–Smirnov normal test was performed on the measurement data. Means ± standard deviations were used for the statistical description of the variables that conformed to a normal distribution, and percentages (%) was used for counting data. An independent sample t-test was used to compare the measurement data. The chi-squared test was used for the comparison of the counting data. All the statistical tests were performed using bilateral tests with alpha = 0.05.

## Data Availability

Data are available upon reasonable request by email (shaohailin1988@sohu.com).
